# Effect of ArtinM on Human Blood Cells During Infection With *Paracoccidioides brasiliensis*

**DOI:** 10.3389/fmicb.2018.00867

**Published:** 2018-05-04

**Authors:** Luciana P. Ruas, Livia M. Genaro, Amauri S. Justo-Junior, Lilian O. Coser, Lívia F. de Castro, Plinio Trabasso, Ronei L. Mamoni, Maria-Cristina Roque-Barreira, Maria-Heloisa S. L. Blotta

**Affiliations:** ^1^Department of Clinical Pathology, School of Medical Sciences, State University of Campinas (UNICAMP), Campinas, Brazil; ^2^Department of Internal Medicine, School of Medical Sciences, State University of Campinas (UNICAMP), Campinas, Brazil; ^3^Department of Morphology and Basic Pathology, Faculty of Medicine of Jundiaí, Jundiaí, Brazil; ^4^Department of Cell and Molecular Biology, Medical School of Ribeirão Preto, University of São Paulo, Ribeirão Preto, Brazil

**Keywords:** *Paracoccidioides*, paracoccidioidomycosis, ArtinM, fungal infection, immune modulation, lectin, neutrophils

## Abstract

Infections caused by fungi are prominent in our environment and can be potentially fatal. paracoccidioidomycosis (PCM), caused by fungi of the *Paracoccidioides* genus, is the most frequent systemic mycosis in Brazil and the main cause of death among immunocompetent individuals. The antifungal therapy for PCM is usually effective but side effects and relapses are often reported. The latter could be avoided with alternative or complementary therapies aimed at boosting the immune response to combat this pathogen. Recent reports have pointed at the importance of an effective cellular immune response, with the participation of Th1 cells, in the resistance to and control of *Paracoccidioides* infection. The ArtinM lectin, extracted from jackfruit (*Artocarpus heterophyllus*) seeds, exhibits immunomodulatory activity against several intracellular pathogens, including *Paracoccidioides brasiliensis*, by promoting the development of a Th1 immune response. The aim of this work was to characterize the effect of ArtinM on peripheral blood cells of patients with PCM and on those of control individuals infected with fungal yeasts cells *in vitro*. Our results demonstrate that ArtinM activates human neutrophils *in vitro*, leading to an increase in cytokine production and CD54 expression. ArtinM activated *P. brasiliensis*-infected neutrophils from both healthy individuals and patients with PCM. This activation was not dependent on the dectin-1 receptor, because pre-incubation with laminarin, a dectin-1 receptor blocker, did not reverse the activated state of the cells. ArtinM also stimulated human peripheral blood mononuclear cells to secrete pro-inflammatory Th1-related cytokines, which are protective against *Paracoccidioides* infection. These data support the immunostimulatory action of ArtinM and encourage new studies using the lectin for the immunotherapy of PCM.

## Introduction

Paracoccidioidomycosis (PCM) is a systemic mycosis that affects tropical and subtropical regions of Latin America. In addition to Brazil, where most cases are reported, Argentina, Venezuela, Colombia, and Ecuador also have a high incidence of PCM (Brummer et al., 1993; Marques, 2003; Restrepo et al., 2012). The disease can affect both children and adults, and is caused by dimorphic fungi of the *Paracoccidioides* genus, such as *Paracoccidioides brasiliensis*, which is the best studied species (Teixeira et al., 2009; Teixeira Mde et al., 2014). The disease can be localized or disseminated, involving mainly the lungs, skin, mucosa, lymph nodes, and central nervous system (Borges-Walmsley et al., 2002).

Paracoccidioidomycosis represents an important public health problem because of its high incapacitating ability and the high premature mortality it causes, especially in rural populations, where PCM is a major cause of death among immunocompetent individuals (Bagagli et al., 2006; Marques, 2012). PCM is characterized by a suppression of the cellular immune response in susceptible individuals, and by the formation of loose and compact granulomas in susceptible and resistant mice, respectively (Calich et al., 1998). Several studies, both in mice and humans, have shown that an efficient immune response against *P. brasiliensis* requires the activation of macrophages and Th1 lymphocytes (Benard et al., 1997, 2001; Calich and Kashino, 1998; Oliveira et al., 2002; Mamoni and Blotta, 2006; Calich et al., 2008).

Although antifungal therapy is being used with success in mycosis caused by dimorphic fungi, the toxicity associated to the therapy constitutes a problem (Bates et al., 2001; Nett and Andes, 2016). For instance, due to its low cost, sulfamethoxazole/trimethoprim is the most common choice for the treatment of PCM in low-income countries, and these, coincidentally, are the major endemic areas. Although this combination is synergistic against the fungus, it has the potential for myelotoxicity, since the drugs sequentially inhibit folate synthesis (Bellmann and Smuszkiewicz, 2017). Itraconazole, a triazole derivate, exhibits the potential for hepatotoxicity, and this might be an issue when treating patients with alcoholism, a very common comorbidity among PCM patients. Furthermore, at least in Brazil, itraconazole is marketed only as tablets with poor or erratic absorption by the gastrointestinal tract (Bellmann and Smuszkiewicz, 2017). Lastly, amphotericin B, a polyene antifungal drug occasionally used for treating life-threatening clinical manifestations of PCM, is well known to be associated with nephrotoxicity, particularly with the use of the deoxycholate formulation (Bellmann and Smuszkiewicz, 2017). In addition, PCM requires a prolonged treatment, usually of more than 1 year, and 20% of the patients present with sequelae, relapse, or complications of the disease (Martinez, 2010). Therefore, more research is needed to develop adjuvant therapies that decrease treatment length and toxicity. One such strategy could be the use of immunotherapies to boost the host immune response to overcome the fungus (Romani, 2011; Ruas et al., 2012; Kullberg et al., 2014).

Lectins are sugar-binding proteins found in virtually all organisms, from viruses to humans (Sharon, 2008). They are involved in several biological activities including adhesion of pathogens to host cells, induction of leukocyte activation and migration, and induction of cytokine production (Sharon and Lis, 2004). Plant lectins have been investigated for decades in biomedical research and are the best studied lectins (Souza et al., 2013). Several plant lectins are considered immunomodulatory agents with high biotechnological potential (Reis et al., 2008; Afonso-Cardoso et al., 2011; de Oliveira et al., 2013; Poiroux et al., 2017). The functionality of these molecules derives from their interaction with glycosylated receptors on the cell surface, which triggers intracellular signaling cascades that culminate in a range of biological responses, including production of cytokines, resulting in an efficient immune response against tumors and microbes (da Silva Correia and Ulevitch, 2002; Unitt and Hornigold, 2011).

ArtinM, obtained from the seeds of *Artocarpus heterophyllus*, is a D-mannose binding lectin with immunomodulatory properties (Souza et al., 2013). ArtinM interacts with TLR2 on macrophages and dendritic cells, resulting in IL-12 production and a polarization of the immune response to a Th1-type (Mariano et al., 2014). Indeed, ArtinM administration to mice leads to protection against several intracellular pathogens, including *Leishmania major* (Panunto-Castelo et al., 2001), *Leishmania amazonensis* (Teixeira et al., 2006), *P. brasiliensis* (Coltri et al., 2008, 2010), *Neospora caninum* (Cardoso et al., 2011), and *Candida albicans* (Loyola et al., 2012). Specifically, *P. brasiliensis*-experimentally infected mice that received ArtinM, either as a prophylactic or therapeutic regimen, had reduced fungal loads in organs and an increased production of immune mediators, such as IL-12 and TNF-α which resulted in protection of mice against the infection (Coltri et al., 2008, 2010). In addition, ArtinM has been reported to have an effect on human cells such as mast cells and neutrophils (Moreno et al., 2003; Pereira-da-Silva et al., 2006; Toledo et al., 2009; Barbosa-Lorenzi et al., 2011, 2016); however, these studies did not include any pathogens or diseases. Therefore, our aim was to evaluate the potential immunostimulatory effect of the lectin ArtinM on cells from PCM patients.

## Materials and Methods

### Donors

Peripheral venous blood was collected from healthy individuals and from PCM patients who attended the University Clinical Hospital of UNICAMP, Campinas, Sao Paulo, Brazil. The diagnosis of PCM was established by identifying the fungus through direct examination, biopsy, or both. The study only included PCM patients with active disease before treatment started or within the first month of treatment. For some assays, blood cells (peripheral blood mononuclear cells – PBMCs or neutrophils) from healthy individuals (controls) were infected with *P. brasiliensis* viable yeasts *in vitro*. Participants were informed about the study and voluntarily signed an informed consent form, as established by the Brazilian National Research Ethics Committee CEP/CONEP (# 574 507).

### Fungi

The highly virulent Pb18 isolate was used throughout the study, as previously described (Kashino et al., 1985). Yeast cells maintained in Fava-Netto medium at 37°C were subcultured weekly and used after 5 days of culture. To maintain the virulence, consecutive passages in mice were carried out by intravenous infection followed by recovery of the fungal isolate from mice tissues. Cells were suspended in phosphate-buffered saline (PBS pH 7.2) and homogenized with glass beads in a Vortex homogenizer to obtain individual cells. Yeast viability was determined by the trypan blue exclusion test and only suspensions with more than 85% of viable cells were used. The opsonization of *P. brasiliensis* yeast cells was carried out by incubating the cells with serum from PCM patients for 10 min at room temperature. The concentration of yeast cells used in each set of experiments was determined as needed.

### ArtinM Preparations

ArtinM lectin was isolated from *A. heterophyllus* seeds and purified by sugar affinity chromatography as previously described (Santos-de-Oliveira et al., 1994).

### Isolation of PBMCs and Polymorphonuclear Cells (PMNs)

Peripheral blood of PCM patients and healthy controls was collected in tubes containing sodium heparin. PBMCs were isolated using Ficoll-Hypaque^®^ (GE Healthcare, United Kingdom) density gradient centrifugation. The buffy coat containing PMNs was transferred to a 15-mL falcon tube, resuspended in red-cell lysis buffer, and incubated at room temperature for 10 min. After washing, the number and viability of PBMCs and neutrophils were estimated by trypan blue exclusion. Cells were plated in 24-well plates with RPMI (Gibco/Thermo Fisher Scientific, Waltham, MA, United States) supplemented with 10% inactivated fetal calf serum, 100 U/mL gentamicin, 2 mM L-glutamine, and 1 mM sodium pyruvate (all from Sigma-Aldrich, St Louis, MO, United States). Cells were incubated with different stimuli for different times as described below.

### Blood Cell Stimulation

Freshly isolated neutrophils and PBMCs were stimulated with ArtinM (2.5 or 5 μg/mL), as previously described (Toledo et al., 2009; Ricci-Azevedo et al., 2016). PMA (50 ng/mL), LPS (1 μg/mL), and PHA (10 μg/mL) were used as positive controls. For some assays, neutrophils were pre-incubated for 30 min with 1 mg/mL of laminarin (β-[1→3]-glucan soluble polymer, obtained from *Laminaria digitata*, Invitrogen, San Diego, CA, United States) to block the dectin-1 receptor (Brown et al., 2002). For the *in vitro* infection assays, cells were incubated with *P. brasiliensis* yeasts (yeast:neutrophil ratio of 1:10) in the presence or absence of ArtinM.

Cells were maintained at 37°C for 4–18 h (neutrophils) or 24–48 h (PBMCs), followed by collection of the supernatants for cytokine determination by ELISA according to the manufacturer’s instructions (R&D Systems, Minneapolis, MN, United States). In addition, neutrophil pellets were collected for the analysis of CD54 expression by FACS.

### Purification of CD14^+^ Cells and Differentiation Into Macrophages

After PBMC isolation, CD14^+^ cells were purified using positive isolation kits, according to the manufacturer’s instructions (MACs; Miltenyi Biotec, Germany). The purity of cell separation was evaluated by flow cytometry (>95% purity). Macrophages were differentiated from the CD14^+^ cells with GM-CSF (50 ng/mL; Biolegend, San Diego, CA, United States) for 5 days and maintained at 37°C (5% CO_2_) in RPMI 1640 supplemented medium. After 7 days, macrophage differentiation was confirmed by microscopy and by the expression of markers as determined by flow cytometry (data not shown).

### Phagocytosis Assays

*Paracoccidioides brasiliensis* yeast cells were labeled with CFSE (1 μM, Sigma-Aldrich) for 5 min. Neutrophils and macrophages were stimulated with ArtinM (2.5 μg/mL) and CFSE-*P. brasiliensis* yeast cells (ratio of 1:5) for 4 h. As a positive control, we used FITC-conjugated Dextran-40 (1 mg/mL, Sigma-Aldrich). The cells were fixed with 2% formaldehyde and analyzed by flow cytometry.

For the killing assays, *P. brasiliensis* yeast cells were incubated with neutrophils or macrophages (ratio of 1:50) for 24 h at 37°C (5% CO_2_). After incubation, neutrophils and macrophages were collected and washed with sterile distilled water to promote cell lysis. The suspension (100 μL) was spread on BHI agar plates supplemented with 4% normal horse serum, 5% *P. brasiliensis* growth factor, and 0.5% gentamicin. A control culture, containing only *P. brasiliensis* yeast cells, underwent the same procedures as the experimental cultures. The number of colony forming units (CFUs) was determined on the 7th and 15th days of culture, and the percentage of killing (fungicidal capacity of cells) was calculated using the formula:

$$\%\text{killing}\;\text{=}\;[1-\frac{\text{CFU number in experimental plates}}{\text{CFU number in control plates}}]\;\times \;100$$

### Flow Cytometry

Cell pellets (approximately 5 × 10^5^ cells) of neutrophils cultured in the presence of ArtinM were incubated for 20 min at 4°C with anti-CD54-PE and anti-CD16-APC antibodies (Biolegend), washed, and fixed with 2% formaldehyde. Labeled and fixed cells were kept in the refrigerator and protected from light, until signal acquisition was performed using a BD-FACSCalibur^®^ or FACSVerse^®^ flow cytometer (BD Biosciences, San Jose, CA, United States). Analysis of the dot-plot or mean fluorescence intensity (MFI) was performed using the FCS Express program (De Novo Software, Glendale, CA, United States).

### Statistical Analysis

Statistical analysis was performed using GraphPad InStat^®^ version 6 (GraphPad Software, San Diego, CA, United States). Normality of data was assessed using the Kolmogorov–Smirnov test. Differences between the averages of the various stimuli were assessed by ANOVA for repeated measures followed by Tukey’s *post hoc* test. For comparisons of the same parameter between different groups, the *t*-test was used. *P* values of less than 0.05 were considered statistical significant.

## Results

### ArtinM Activates Human Neutrophils Stimulated With *P. brasiliensis* Yeast Cells *in Vitro*

**FIGURE 1:**
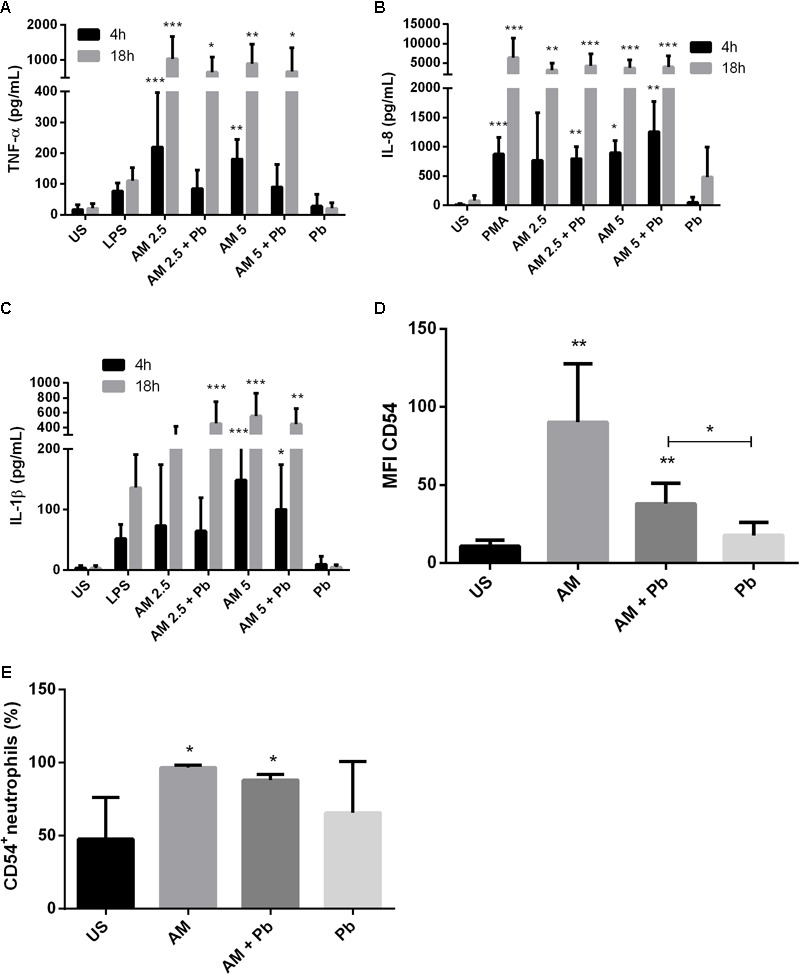
ArtinM induces activation and cytokine production in neutrophils infected with *P. brasiliensis* yeast cells *in vitro*. Neutrophils obtained from the peripheral blood of healthy individuals (*n* = 10) were maintained *in vitro* in the presence of the indicated stimuli, for 4 and 18 h. Culture supernatants were collected and used for cytokine quantification by ELISA (**A**: TNF-α, **B**: IL-8, **C**: IL-1β). Cell pellets were assayed for CD54 expression **(D)** and frequency **(E)** by FACs. AM: ArtinM, Pb: *P. brasiliensis* yeast (yeast:neutrophil ratio of 1:10), US: unstimulated. ^∗^*P* ≤ 0.05, ^∗∗^*P* ≤ 0.01, ^∗∗∗^*P* ≤ 0.001 compared to US.

Neutrophils from healthy individuals were stimulated *in vitro* with *P. brasiliensis* yeast cells, ArtinM, or both, for different times, and cytokine production in supernatants was determined as outlined in Section “Materials and Methods.” An increased TNF-α production was observed in neutrophils infected with *P. brasiliensis* after 18 h of incubation with ArtinM (**Figure 1A**), while induction of TNF-α production in uninfected neutrophils occurred much earlier (4 h).

Previous reports had shown that ArtinM stimulation leads to IL-8 production in human neutrophils (Toledo et al., 2009; Ricci-Azevedo et al., 2016). Here, we evaluated whether this also occurs during PCM. Our results showed that ArtinM induces an early IL-8 production in neutrophils infected with *P. brasiliensis* (**Figure 1B**). After 18 h, the production of IL-8 induced by ArtinM reached similar levels to those induced by PMA stimulation, even in the presence of *P. brasiliensis*.

Because ArtinM stimulation of neutrophils led to the production of pro-inflammatory cytokines, we quantified IL-1β production in neutrophils infected *in vitro* with *P. brasiliensis*. We found that ArtinM induced IL-1β production in both infected and uninfected neutrophils. After 18 h, we found that even the lowest concentration of lectin (2.5 μg/mL) led to the production of IL-1β in cells infected with *P. brasiliensis* (**Figure 1C**).

Next, we evaluated whether activation of these cells was also reflected by expression of CD54/ICAM-1, a molecule involved in neutrophil adhesion and activation. ArtinM induced a ninefold increase in CD54/ICAM-1 expression on neutrophils (**Figure 1D**). The addition of 2.5 μg/mL of the lectin to neutrophils doubled the percentage of CD54^+^ neutrophils, even in cells infected with viable yeasts (**Figure 1E**).

### ArtinM Activates Neutrophils From PCM Patients

**FIGURE 2:**
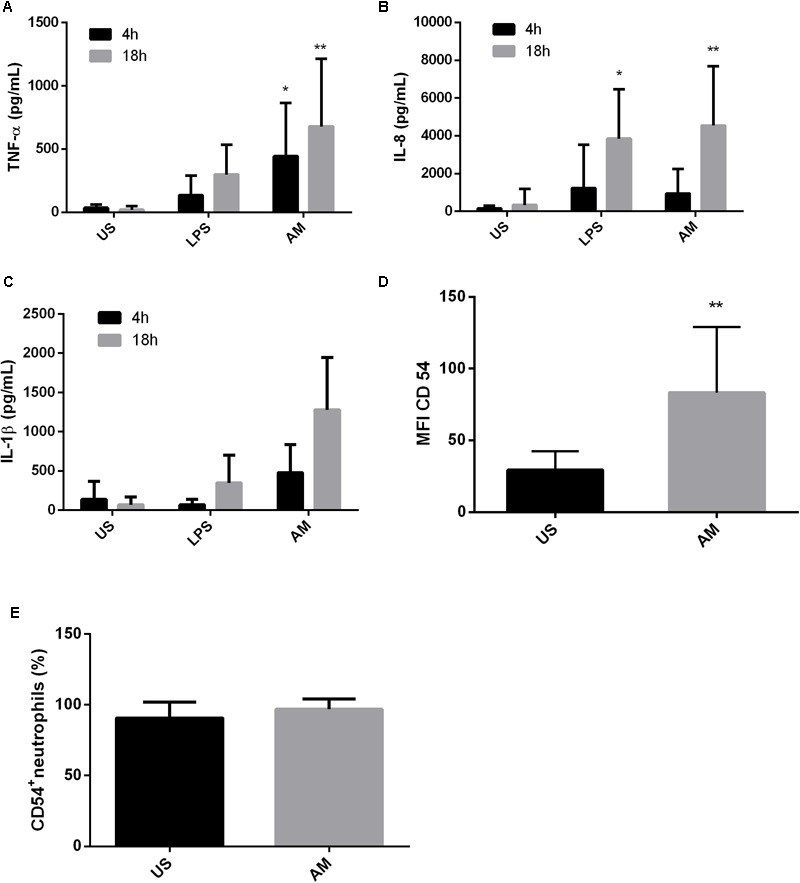
ArtinM induces activation and cytokine production in neutrophils of PCM patients. Neutrophils obtained from the peripheral blood of patients with paracoccidioidomycosis (*n* = 10) were maintained *in vitro* with the indicated stimuli for 4 and 18 h. Culture supernatants were collected and used for cytokine quantification by ELISA (**A**: TNF-α, **B**: IL-8, **C**: IL-1β). Cell pellets were assayed for CD54 expression **(D)** and frequency **(E)** by FACs. AM: ArtinM, US: unstimulated. ^∗^*P* ≤ 0.05, ^∗∗^*P* ≤ 0.01 compared to US.

Next, we assessed if the beneficial effects promoted by ArtinM during *in vitro* fungal infection were reproduced in individuals with PCM. To this end, blood from patients with active disease was collected. As shown in **Figure 2**, ArtinM stimulated the production of TNF-α and IL-8 in neutrophils of PCM patients after 18 h of incubation. Importantly, even though the basal levels of IL-8 produced by neutrophils of PCM patients were already high, ArtinM was still able to stimulate these neutrophils, leading to a sevenfold rise in IL-8 production compared to unstimulated cells (**Figure 2B**). In addition, ArtinM enhanced TNF-α production in neutrophils of PCM patients leading to a 6- and 100-fold increase after 4 and 18 h of incubation, respectively (**Figure 2A**). Moreover, IL-1β production was also induced in neutrophils of PCM patients after 18 h of incubation with ArtinM compared to unstimulated cells, although this difference was not statistically significant (**Figure 2C**).

Finally, we evaluated the effect of ArtinM stimulation on the frequency and expression of the CD54 molecule in neutrophils of PCM patients. Similar to that observed in cells from healthy individuals infected *in vitro*, an increase in the expression of CD54 on neutrophils from PCM patients was observed following ArtinM stimulation (**Figure 2D**). Almost 100% of these neutrophils were already CD54^+^; therefore, an increase in this population could not be detected after the addition of the lectin (**Figure 2E**).

### Neutrophil Activation by ArtinM Is Independent of β-Glucan Receptor Dectin-1

**FIGURE 3:**
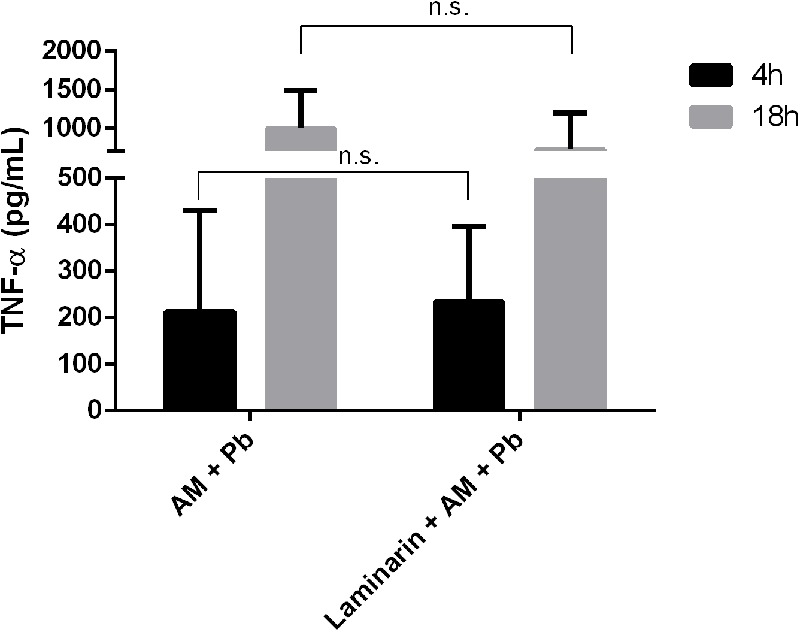
Dectin-1 blockage does not alter TNF-α production induced by ArtinM in infected neutrophils. Neutrophils were obtained from the peripheral blood of healthy individuals (*n* = 7), pre-incubated with laminarin for 30 min, and incubated *in vitro* with the indicated stimuli, for 4 and 18 h. Culture supernatants were collected and used for TNF-α quantification by ELISA. AM, ArtinM; Pb, *P. brasiliensis* yeast (yeast:neutrophil ratio of 1:10); ns: non-significant.

Dectin-1 is involved in the production of TNF-α by cells in response to fungal recognition (Brown et al., 2003). Therefore, we evaluated if dectin-1 was also involved in cell activation in response to ArtinM. To this end, cells were pre-treated with the dectin-1 antagonist laminarin for 30 min, followed by incubation with ArtinM, and measurement of activation parameters as before. Pre-incubation of neutrophils from healthy individuals with laminarin did not affect TNF-α production in response to ArtinM (**Figure 3**), neither was affected the percentage of CD54^+^ cells or CD54 expression in neutrophils infected *in vitro* with *P. brasiliensis* or in neutrophils from PCM patients (data not shown). These data suggest that activation of infected neutrophils by ArtinM does not depend on the receptor dectin-1.

### ArtinM Activates PBMCs Stimulated *in Vitro* With *P. brasiliensis* Yeast Cells

**FIGURE 4:**
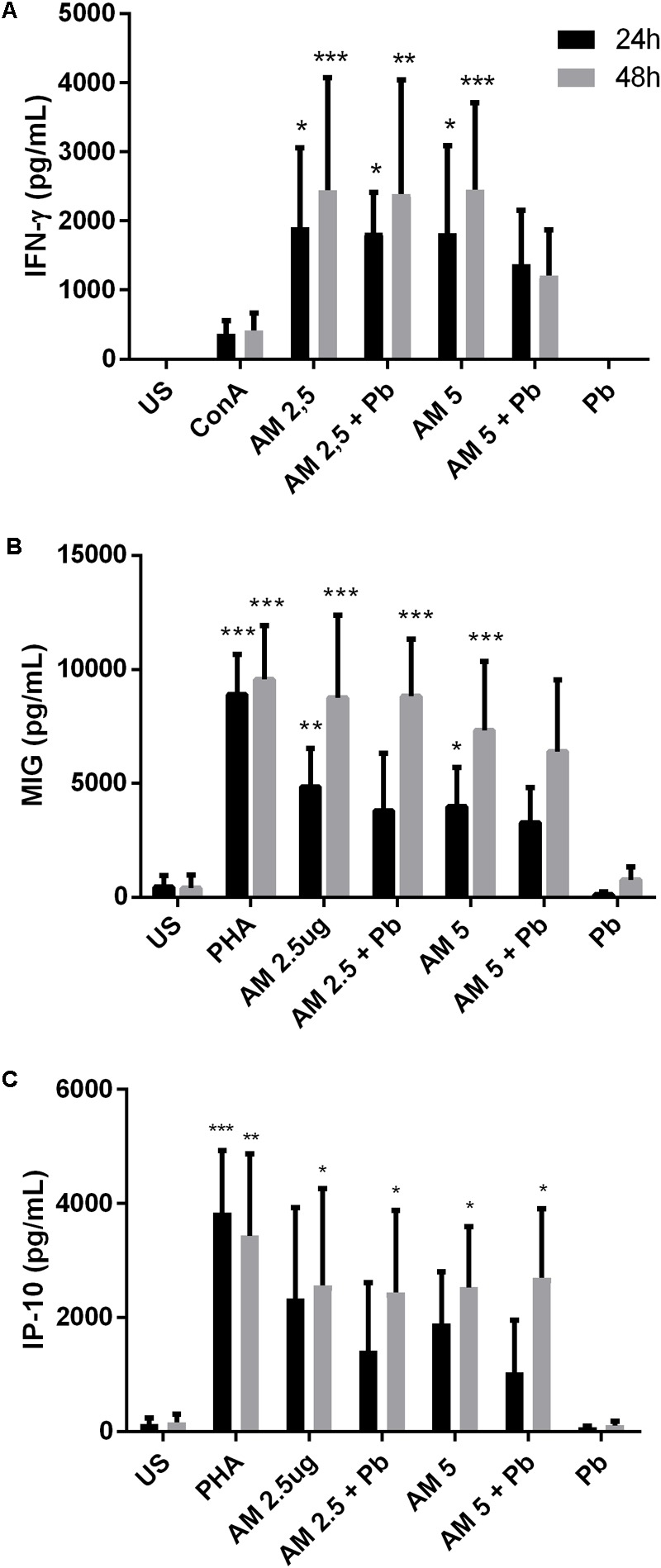
ArtinM induces cytokine production in PBMCs infected in vitro with *P. brasiliensis* yeasts. PBMCs obtained from the peripheral blood of healthy individuals (*n* = 7) were maintained *in vitro* with the indicated stimuli, for 24 and 48 h. Culture supernatants were collected and used for cytokine quantification by ELISA (**A**: IFN-γ, **B**: MIG, **C**: IP-10). AM: ArtinM, Pb: *P. brasiliensis* yeast (yeast:PBMC ratio of 1:10), US: unstimulated. ^∗^*P* ≤ 0.05, ^∗∗^*P* ≤ 0.01, ^∗∗∗^*P* ≤ 0.001 compared to US.

Next, we wanted to assay the effect of the ArtinM lectin on other blood cell types. To this end, we used PBMCs from healthy individuals infected *in vitro* with *P. brasiliensis* yeasts. ArtinM induced an increased production of the Th1 cytokines IFN-γ (**Figure 4A**), MIG/CXCL9 (**Figure 4B**), and IP10/CXCL10 (**Figure 4C**) in PBMCs of healthy individuals regardless of whether they had been infected with *P. brasiliensis* yeasts. The increase in cytokine production was detected for the two lectin concentrations tested (2.5 and 5 μg/mL). MIG and IP10 chemokines are related to the Th1-type immune response, because they are induced by IFN-γ (Luster et al., 1985; Farber, 1990). Polarization to a Th1 response has already been suggested to be the mechanism of action of ArtinM against several pathogens in experimental models (Panunto-Castelo et al., 2001; Teixeira et al., 2006; Coltri et al., 2008, 2010; Cardoso et al., 2011; Custodio et al., 2011).

### ArtinM Activates PBMC From PCM Patients

**FIGURE 5:**
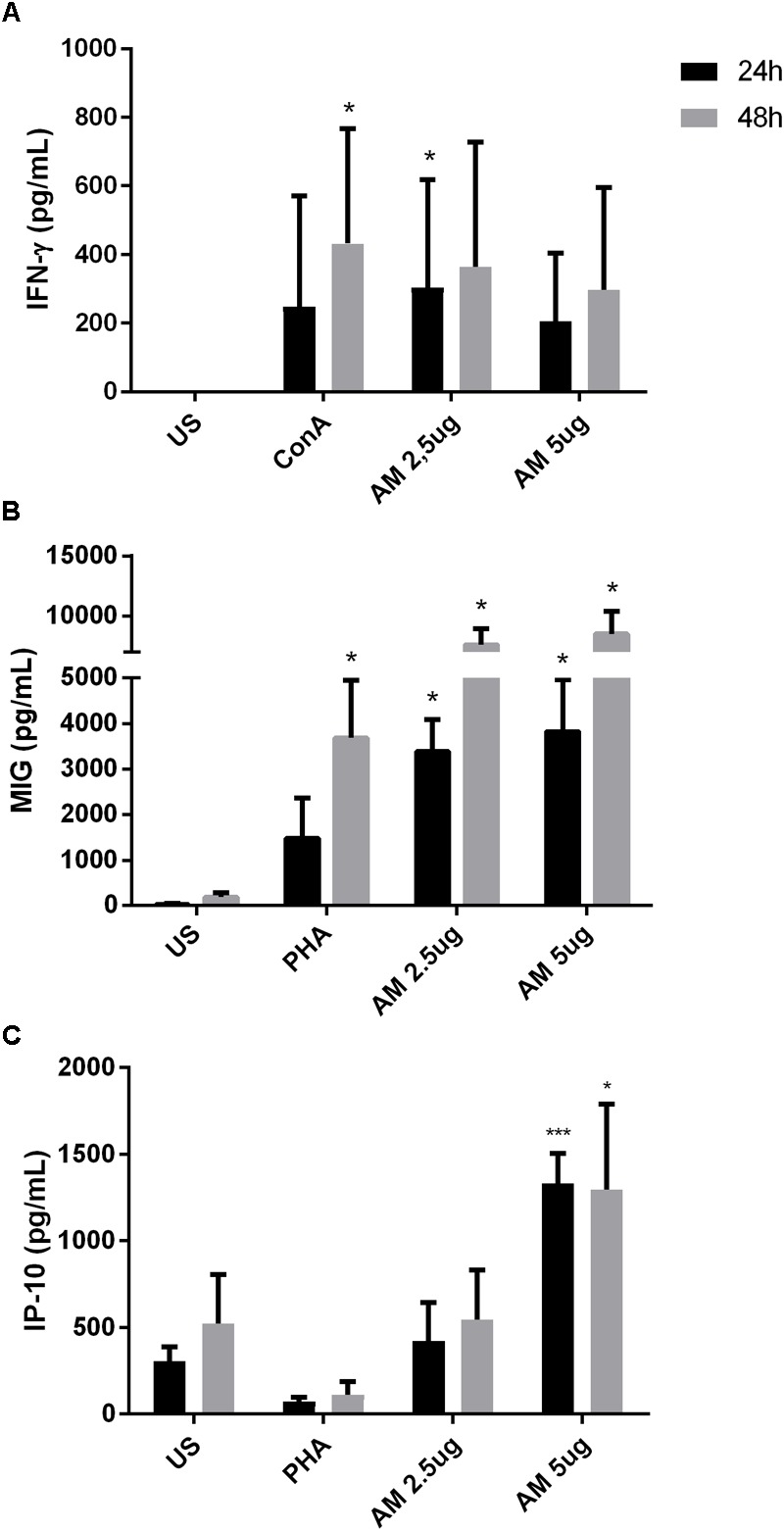
ArtinM induces cytokine production in PBMCs of PCM patients. PBMCs obtained from the peripheral blood of patients (*n* = 8) with PCM were maintained *in vitro* with the indicated stimuli, over 24 and 48 h. Culture supernatants were collected and used for cytokine quantification by ELISA (**A**: IFN-γ, **B**: MIG, **C**: IP-10). AM: ArtinM, Pb: *P. brasiliensis* yeast (yeast:PBMC ratio of 1:10), US: unstimulated. ^∗^*P* ≤ 0.05, ^∗∗^*P* ≤ 0.01, ^∗∗∗^*P* ≤ 0.001 compared to US.

To evaluate the effect of ArtinM during human PCM, venous blood from PCM patients was collected and PBMCs were stimulated with ArtinM for 24 and 48 h. Cytokine production was quantified in the supernatants as described in the Materials and Methods section. Consistent with the results from *in vitro* infection of healthy PBMCs, ArtinM stimulation of cells from PCM patients induced an increased production of IFN-γ (**Figure 5A**), MIG (**Figure 5B**), and IP-10 (**Figure 5C**). Our results confirm that the Th1 polarization induced by ArtinM during experimental PCM also occurs in human blood cells from PCM patients.

### ArtinM Promotes Increased *P. brasiliensis* Internalization by Neutrophils and Macrophages

**FIGURE 6:**
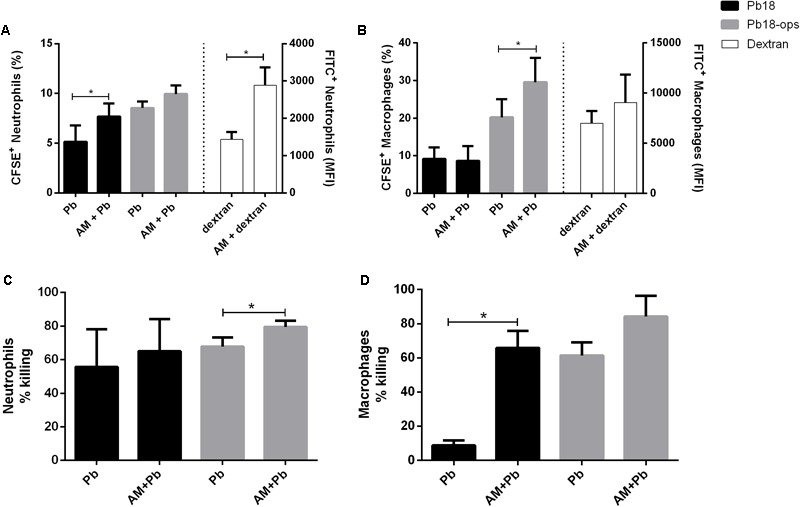
ArtinM promotes increase of *P. brasiliensis* internalization **(A,B)** and killing **(C,D)** by neutrophils and macrophages. Neutrophils and macrophages were maintained *in vitro* with CFSE-Pb18 yeasts in the presence or not of ArtinM, for 4 h. FITC-Dextran was used as positive control. **(A,B)** Cell pellets were assayed for CFSE/FITC frequency and expression by FACs. **(C,D)** neutrophils and macrophages were lysed, yeast CFU was counted, and the percentage of fungicidal activity was calculated as described in Section “Materials and Methods.” AM, ArtinM; Pb, *P. brasiliensis* yeast. ^∗^*P* ≤ 0.05, compared to Pb, in the absence of ArtinM.

We next evaluated whether the activation of cells promoted by ArtinM would improve the phagocytic capacity of neutrophils and macrophages. We incubated CFSE-labeled Pb yeast with cells, with or without ArtinM, and used FITC-dextran as a positive control. As shown in **Figure 6**, ArtinM promoted the internalization of CFSE yeasts cells by both neutrophils and macrophages (**Figures 6A,B**). In macrophages, yeast opsonization was necessary to promote the internalization (**Figure 6B**). Since almost 100% of the neutrophils and macrophages internalized labeled dextran particles, we examined the MFI. As shown in **Figure 6A** (right bars), ArtinM improved the internalization of dextran particles by neutrophils, which might indicate that the lectin would promote the phagocytosis of not only *P. brasiliensis*, but also of other microorganisms,.

Because ArtinM promoted the phagocytosis of Pb18 yeast, we analyzed if the lectin had an effect on the fungicidal capacity of these cells. Indeed, the killing capacity of neutrophils and macrophages was higher when these cells were incubated with ArtinM (**Figures 6C,D**).

## Discussion

The immunomodulatory protective role played by the ArtinM lectin in murine PCM encouraged us to study the effect of this lectin in human leukocytes obtained from healthy control individuals and stimulated *in vitro* with *P. brasiliensis* yeast, and from PCM patients. One of the most striking features of the immune response in PCM is the immunosuppression of the cell-mediated response observed in patients presenting the active form of the disease. In fact, the degree of immunosuppression is associated with the severity of the disease (Musatti et al., 1976; Shikanai-Yasuda et al., 1992; Benard et al., 1997, 2001).

ArtinM stimulates a Th1 immunity conferring resistance to some intracellular pathogens (Ruas et al., 2012; Souza et al., 2013). We reasoned that the study of immune cells during *P. brasiliensis* infection in the presence of immunomodulatory agents such as ArtinM would help to better understand the disease and to develop new treatments. For instance, the analysis of the response of blood cells to therapeutic agents, in terms of cytokine production and expression of activation markers present on the cell surface, provides important information concerning the immunomodulatory activity of those therapeutic agents (Arvå and Andersson, 1999; Collins, 2000; Reddy et al., 2004).

Even with the recent development of less toxic antifungal drugs, fungi still represent the leading cause of death in intensive care units (Shoham and Marwaha, 2010; Paramythiotou et al., 2014). It is known that host defense mechanisms influence the manifestation and severity of fungal diseases (Romani, 2004), and several reports have shown the importance of adjunctive immunotherapies for the treatment of these infections (Kullberg et al., 2014; Cutino-Moguel et al., 2017; Posch et al., 2017). Several plant and pathogen lectins have immunomodulatory properties with potential pharmaceutical applications. The recognition of cell surface receptors by lectins accounts for their immunomodulatory properties; it has been reported that the plant lectin ArtinM acts as a TLR agonist, thereby improving the host immune response (Da Silva and Correia, 2014; Ricci-Azevedo et al., 2017). ArtinM interaction with pattern recognition receptors, such as TLRs, triggers cell signaling events that culminate in the production of IL-12 and consequently, Th1 cytokines (Ricci-Azevedo et al., 2017).

TNF-α plays a central role in the host defense against microorganisms as demonstrated by the exacerbation or onset of infectious diseases, such as tuberculosis, that occurs in patients with inflammatory diseases that are treated with anti-TNF antibodies (Lin and Ottenhoff, 2008). TNF-α augments the neutrophil oxidative burst in response to a wide range of stimuli, including fungi, bacterial, and protozoan infections (Ferrante, 1992). It has been extensively reported that TNF-α activates both human and murine leukocytes to exert their fungicidal activities against *P. brasiliensis* (Brummer et al., 1989; Gonzales et al., 2003; Rodrigues et al., 2007; Moreira et al., 2008). Loyola et al. (2012) reported an ArtinM-mediated TNF-α-induction in murine macrophages. Importantly, TNF-α has chemotactic activity for immune cells, including neutrophils (Ming et al., 1987), thereby contributing to the amplification of the activation of the immune system.

Dectin-1 is a cell surface receptor present in many cells of the immune system, including neutrophils (Taylor et al., 2002). It recognizes β-glucan, present in the fungal cell wall, and therefore participates in the recognition of many fungal species, including *P. brasiliensis* (Balderramas et al., 2014; Feriotti et al., 2015; Loures et al., 2015; Bachiega et al., 2016). Recently, Romagnolo et al. (2018) demonstrated that this receptor participates in cytokine secretion by monocytes when stimulated *in vitro* with *P. brasiliensis* 265 isolate. Our results show that this receptor is not involved in ArtinM activation of neutrophils infected with Pb18 isolate. We hypothesize that other PRRs might be involved in ArtinM activation of cells during *P. brasiliensis* infection and this is currently under investigation in our laboratory.

It has been previously demonstrated that the ArtinM lectin activates human neutrophils (Toledo et al., 2009; Ricci-Azevedo et al., 2016). These activated neutrophils undergo some phenotypic and functional changes such as increased phagocytosis and lysis, L-selectin shedding, and production of inflammatory mediators, such as IL-8 (Toledo et al., 2009). Similarly, our results show that ArtinM induces IL-8 production in neutrophils from PCM patients, which could increase the anti-apoptotic effect of TNF-α (Cowburn et al., 2004) as well as amplify the immune response. In a recent paper, Ricci-Azevedo et al. (2016) showed by several methods that ArtinM prolongs the survival of naive neutrophils. In the same report, the authors demonstrated that neutrophils contribute to ArtinM-induced protection against *L. major*. Specifically, neutrophils infected with the protozoan and stimulated with ArtinM had an enhanced leishmanicidal activity, and produced high levels of TNF-α and IL-1β (Ricci-Azevedo et al., 2016). Likewise, our data show that activation of neutrophils by ArtinM is not inhibited by *P. brasiliensis* infection *in vitro*, and similar results were obtained with neutrophils from PCM patients.

IL-1β participates in a variety of inflammatory events including production of other cytokines such as IL-6, which contributes to the maintenance of inflammation (Dinarello, 1996). Microbial agents stimulate phagocytic cells to produce IL-1β in its inactive form (pro-IL-1β). The cleavage of pro-IL-1β to its active form, in turn, requires the participation of the inflammasome, a cytosolic multiprotein complex that acts upon recognition of endogenous and pathogenic molecules (Martinon et al., 2002; Franchi et al., 2010). Importantly, activation of the inflammasome and IL-1β production has been associated with resistance to fungal diseases (Vonk et al., 2006; Gross et al., 2009; Joly et al., 2009). In this regard, Tavares et al. (2013) have shown that murine dendritic cells produce IL-1β via the NLRP3 inflammasome in response to *P. brasiliensis*. In addition, several researchers have reported the important role of inflammasome activation and of the generation of an IL-1β inflammatory response during infection with *P. brasiliensis* (Feriotti et al., 2015; Ketelut-Carneiro et al., 2015). Notably, patients with the acute form of PCM present with high serum levels of cytokines of the IL-1 family, including IL-1β (Alves et al., 2018). The increased production of IL-1β in *P. brasiliensis*-infected neutrophils stimulated with ArtinM suggests a possible role of the lectin in inflammasome activation and, hence, in the generation of a protective response against *P. brasiliensis*.

Previously, Loyola et al. (2012) reported that ArtinM promoted an increase in the phagocytosis of *C. albicans* by murine macrophages. ArtinM was also described as a neutrophil activator and, among other functions, induced the phagocytosis of zymosan particles and *Listeria monocytogenes* (Toledo et al., 2009). Similarly, our results show that ArtinM promotes an increase in the internalization of *P. brasiliensis* yeast by neutrophils and macrophages. However, it has been reported that ingested *P. brasiliensis* can multiply inside macrophages, but this multiplication can be inhibited when the cells are activated (Moscardi-Bacchi et al., 1994). Once we noted that ArtinM activates neutrophils in the presence of Pb yeast, we also evaluated the fungicidal capacity of these cells stimulated with the lectin. Indeed, ArtinM improved the *P. brasiliensis*-killing capacity of neutrophils and macrophages *in vitro*. The increase in phagocytosis promoted by ArtinM may be related to the high production of cytokines, such as TNF-α and IFN-γ by these cells when in the presence of the lectin (**Figures 1, 2, 4, 5**). In fact, several authors have reported the importance of cytokines in the activation of phagocytic cells and killing of *P. brasiliensis* yeast (Cano et al., 1992; Moscardi-Bacchi et al., 1994; Calvi et al., 2003; Carmo et al., 2006; Rodrigues et al., 2007; Acorci-Valério et al., 2010). Similar to ArtinM, a plant lectin, natural components, such as propolis, have been shown to improve the fungicidal activity of macrophages against *P. brasiliensis* (Murad et al., 2002).

Immunoregulation in PCM is clearly related to the ability of the host to develop a cellular immune response. It has been reported that healthy individuals who live in PCM-endemic areas but do not develop the disease, in spite of being in contact with the fungus, develop a Th1 immune response, with the formation of compact granulomas, leading to the retention and subsequent elimination of the yeasts cells (Mamoni and Blotta, 2005; Benard, 2008). In contrast, in patients with active disease, the cellular Th1 immune response is suppressed, while there is a production of anti-inflammatory and regulatory cytokines, which are associated with fungal evasion and disease dissemination (Franco et al., 1987; Benard et al., 2001; Neworal et al., 2003; Mamoni and Blotta, 2006; Ferreira et al., 2010). Our results show that ArtinM activates neutrophils and PBMCs from PCM patients to secrete cytokines and chemokines, which may amplify the immune response against the fungus. In this regard, da Silva et al. (2014) have shown that ArtinM activates murine CD4^+^ T cells leading to IL-2 production and T-cell proliferation. The authors suggested that ArtinM interaction with T cells provides an additional mechanism of adaptive Th1-immune response induction by the lectin, thus enhancing the resulting response. Because PCM patients present a downregulated immune response, it is likely that the ArtinM pro-inflammatory stimulus could act as an adjuvant boosting the host immune response.

We found that neutrophils activated by ArtinM have an increased expression of the CD54 protein, a glycoprotein expressed on the cell surface, which functions in neutrophil migration and activation. Previous studies have shown that CD54 expression is upregulated by inflammatory mediators and cytokines such as TNF-α and IL-1β (Wedi et al., 1996; Wang et al., 1997; Roebuck and Finnegan, 1999). Therefore, the production of cytokines by *P. brasiliensis*-infected neutrophils (both *in vitro* infected and those obtained from PCM patients) stimulated with ArtinM supports the higher expression of CD54 in these cells. Hence, we conclude that ArtinM is able to activate neutrophils during *P. brasiliensis* infection, as demonstrated by an increase in pro-inflammatory cytokines, which is associated with higher CD54 expression. Furthermore, we rule out the involvement of the dectin-1 receptor in ArtinM-induced activation of *P. brasiliensis*-infected neutrophils, because its blockage did not affect TNF-α production or CD54 expression in human neutrophils.

Peripheral blood mononuclear cells are activated during *P. brasiliensis* infection, and produce Th1 cytokines upon ArtinM stimulation, which supports the lectin immunomodulatory function.

The use of the plant lectin in clinical assays requires additional preliminary studies. The aim of our study was to show that the previously described immunostimulatory properties of ArtinM are replicated in human cells, including those from PCM patients, which are known to be unresponsive to several stimulus (Benard et al., 1997, 2001; Oliveira et al., 2002). It has been recently published that administration in naive mice of low doses of ArtinM (2.5 μg/mL) does not have unwanted effects, while administration of high doses is only associated with the development of mild inflammatory infiltrates in tissues (Oliveira Brito et al., 2017). This supports the potential use of the lectin as a therapeutic aid. The research on lectins is encouraging thanks to lectin engineering, a promising tool for expanding protein applications and utilities (Hu et al., 2015). Our results support the studies on the use of ArtinM, or ArtinM-like engineered molecules as an adjuvant therapy for PCM, in order to boost the immune response of the patient, or as an alternative to the current therapy.

## Ethics Statement

The study was approved by the University of Campinas (UNICAMP) Research Ethics Committee (# 574 507).

## Author Contributions

LR: concept and design, data collection, analysis and interpretation of data, *in vitro* experiments, critical revision, and intellectual content. LG, AJ-J, LOC, LFC: *in vitro* experiments and data collection. PT: patients’ recruitment and clinical evaluation. RM: critical revision. M-CR-B: reagents’ supply and critical revision. M-HB: handle funding and supervision, concept and design, analysis and interpretation of data, critical revision, and intellectual content.

## Conflict of Interest Statement

The authors declare that the research was conducted in the absence of any commercial or financial relationships that could be construed as a potential conflict of interest. The handling Editor declared a shared affiliation, though no other collaboration, with one of the authors M-CR-B.
